# Multi-Scale and Shape Constrained Localized Region-Based Active Contour Segmentation of Uterine Fibroid Ultrasound Images in HIFU Therapy

**DOI:** 10.1371/journal.pone.0103334

**Published:** 2014-07-25

**Authors:** Xiangyun Liao, Zhiyong Yuan, Qi Zheng, Qian Yin, Dong Zhang, Jianhui Zhao

**Affiliations:** 1 School of Computer, Wuhan University, Wuhan, Hubei, China; 2 College of Information Science and Technology, Beijing Normal University, Beijing, China; 3 School of Physics and Technology, Wuhan University, Wuhan, Hubei, China; Institute of Automation, Chinese Academy of Sciences, China

## Abstract

**Purpose:**

To overcome the severe intensity inhomogeneity and blurry boundaries in HIFU (High Intensity Focused Ultrasound) ultrasound images, an accurate and efficient multi-scale and shape constrained localized region-based active contour model (MSLCV), was developed to accurately and efficiently segment the target region in HIFU ultrasound images of uterine fibroids.

**Methods:**

We incorporated a new shape constraint into the localized region-based active contour, which constrained the active contour to obtain the desired, accurate segmentation, avoiding boundary leakage and excessive contraction. Localized region-based active contour modeling is suitable for ultrasound images, but it still cannot acquire satisfactory segmentation for HIFU ultrasound images of uterine fibroids. We improved the localized region-based active contour model by incorporating a shape constraint into region-based level set framework to increase segmentation accuracy. Some improvement measures were proposed to overcome the sensitivity of initialization, and a multi-scale segmentation method was proposed to improve segmentation efficiency. We also designed an adaptive localizing radius size selection function to acquire better segmentation results.

**Results:**

Experimental results demonstrated that the MSLCV model was significantly more accurate and efficient than conventional methods. The MSLCV model has been quantitatively validated via experiments, obtaining an average of 0.94 for the DSC (Dice similarity coefficient) and 25.16 for the MSSD (mean sum of square distance). Moreover, by using the multi-scale segmentation method, the MSLCV model’s average segmentation time was decreased to approximately 1/8 that of the localized region-based active contour model (the LCV model).

**Conclusions:**

An accurate and efficient multi-scale and shape constrained localized region-based active contour model was designed for the semi-automatic segmentation of uterine fibroid ultrasound (UFUS) images in HIFU therapy. Compared with other methods, it provided more accurate and more efficient segmentation results that are very close to those obtained from manual segmentation by a specialist.

## Introduction

Uterine fibroids are commonly occurring benign tumors that can trouble females. HIFU therapy, a new type of noninvasive surgery, has been gradually applied to the treatment of uterine fibroids for its safety and effectiveness, reducing pain caused by traditional surgery [1]–[4]. As is widely known, precise segmentation of ultrasound images has always been a problem with regard to image segmentation, and there has not been an ideal solution until now due to the images’ low SNR (signal to noise ratio), weak boundaries and intensity inhomogeneity [5]. More serious noise and blurry boundaries have been observed in images used in HIFU therapy because of water interference in the treatment process. However, segmentation of target regions, whose precision decides the surgery’s final result, is the most critical stage in HIFU therapy. In the meantime, real-time performance is a great advantage of ultrasound-led HIFU systems, and the speed of segmentation of tumor regions greatly impacts the overall process of the surgery [6]–[8]. For these reasons, development of a highly effective and precise image segmentation method for ultrasound images in HIFU therapy is urgently needed.

Active contour modeling has been widely applied in medical image segmentation in recent years as a consequence of its smoothness and closure, and because it can obtain fairly good results [9]–[11]. Active contour modeling, which was first proposed by Kass *et al*. [12], can be classified into two categories: edge-based modeling [12]–[14] and region-based modeling [15]–[18]. The edge-based active contour model adopts the image’s gradient information as an image-based “force” to push the contour toward the target boundary, and achieves a good segmentation result for target regions with clear edges. However, sensitivity to noise in the image and the initial contours [19], the two major drawbacks of this model, exist because gradient information is highly localized image information, so the problem of edge leakage can easily be produced when this model is applied to ultrasound images, such as GVF (Gradient Vectov Flow) [20]. Region-based active contour modeling, in which the driving force is formed using statistical information about the foreground and background regions, applies to image segmentation where the intensity is homogeneously distributed inside regions. The most famous region-based active contour model is the piecewise constant model by Chan and Vese (C-V model) [16], which effectively segments ultrasound images with noise and weak edges as a consequence of not using the images’ gradient information. However, incorrect results can be produced on HIFU ultrasound images with intensity inhomogeneity because the C-V model assumes regions of intensity in the image are homogeneously distributed and utilizes global statistical information [16], [19].

**Figure 1:**
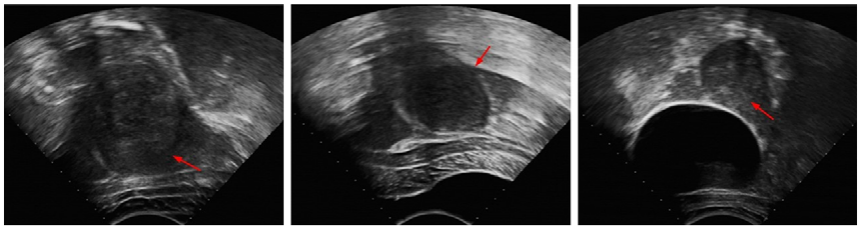
HIFU ultrasound images of uterine fibroids with extremely low SNR, low contrast and weak edges; the red arrows show edges that can easily lead to incorrect segmentation.

To overcome the shortcoming that makes the region-based active contour model difficult to use when segmenting targets with inhomogeneous intensity, localizing the region-based model has attracted interest, and many models using localized information have been proposed. Li *et al*. [21] presented a model where a kernel function was introduced to define local binary fitting energy in a variational level set framework, thus incorporating local grayscale information into a region-based active contour model. Then, Li *et al*. [18] made improvements on this model and studied in depth the selection of kernel functions and the size of localized regions. S. Lankton *et al*. [19] came up with a localizing framework that allowed a region-based energy equation that utilized global information to be rewritten in localized form and thoroughly analyzed the impact of the localizing radius on segmentation results. Zhang *et al*. [22] put forward a similar localized fitting energy, improving calculation efficiency. Later, Wang *et al*. [23] proposed a region-based grayscale level fitting energy combining global and local information with better flexibility. Similarly, Wu *et al*. [24] presented the average misclassification probability (AMP) model, as well as a global-to-local strategy by combining global and local information to enhance segmentation results on complex images. Appia *et al*. [25] introduced a global edge-based constraint into the region-based model. All of these models include local information and are suitable approaches for the ultrasound image segmentation problem. They have better segmentation capacity for images with intensity inhomogeneity than region-based active contour models that use global information, but for UFUS images in HIFU therapy that have seriously low SNR, low contrast and weak edges, as shown in Figure 1, the localized region-based active contour model may still produce incorrect segmentation with boundary leakage or excessive contraction. To solve this problem, we proposed incorporating a shape constraint into the localized region-based active contour model. Meanwhile, we studied adaptive selection of localizing radius that is suitable for segmentation of UFUS images in HIFU therapy to acquire better segmentation results.

As mentioned in [5], for ultrasound images, depending only on edge and region information are usually not sufficient to obtain reliable and precise segmentation. In this case, shape constraint is usually used to improve the segmentation results [26]–[30]. Leventon *et al*. [26] used a level set representation to model the shape prior by computing a principal components analysis (PCA) of training shapes incorporated in level set functions, but the variational formulation was not associated with evolution equation. Chen *et al*. [27] designed a novel variational model that incorporated prior shape knowledge into geometric active contours. Later, Chen presented a method to solve the minimization problem of the coupling of prior shape and intensity profiles for image segmentation [28]. Bresson *et al*. [29] used a space of a given shape in the target region to build a shape energy and used local edge information and global image information at the same time. Recently, Wu *et al*. [30] also combined prior high-level shape information to build a non-parametric statistical shape model and applied it to prostate segmentation. Huang *et al*. [31] focuses on optimization of robust graph-based (RGB) segmentation algorithm to extract breast tumors in ultrasound images more adaptively and accurately. Considering that the benign uterine fibroids usually approximate ellipsoid shapes of different sizes, we take an ellipse as an example to form a shape constraint to segment UFUS images for HIFU therapy. In this paper, we incorporate a shape constraint into a localized region-based level set framework to obtain the desired and accurate segmentation avoiding boundary leakage and excessive contraction.

To reduce the calculation time consumed by segmentation of HIFU ultrasonic images, a multi-scale segmentation method is an effective way to significantly improve the segmentation efficiency in HIFU therapy. Yu *et al*. [32] proposed a novel method for breast mass segmentation based on the level set method and multi-scale analysis. Kim *et al*. [33] introduced a flow-based multi-scale framework for unsupervised surface defect segmentation based on the multi-scale scheme of the phase spectrum of Fourier transform. Zhou *et al*. [34] proposed a new multi-scale saliency detection algorithm based on image patches. Wang *et al*. [35] presented a multi-scale framework for segmentation of ultrasound image based on speckle-reducing anisotropic diffusion and geodesic active contours. All of these previous works utilized multi-scale methods to improve segmentation efficiency by reducing the heavy computational burden in some way. Thus, we propose introducing a multi-scale segmentation method to improve the efficiency of segmentation of the target regions of UFUS in HIFU therapy.

In this work, our contributions are focused on following 3 parts. First, by incorporating a new shape constraint into localized region-based active contouring, the MSLCV model is able to address ultrasound images with substantial noise and weak edges, and even with some missing information. This model provides more precise segmentation, and the shape constraint is universal and easy to use. Second, to optimize the calculation, we analyze and utilize a multi-scale segmentation algorithm to greatly improve the efficiency of segmentation. Third, we have studied the selection of localizing the radius in depth and have designed an adaptive radius size selection function for the segmentation of UFUS images in HIFU therapy. Meanwhile, considering that the proposed method relies on initialization, we put forward some corresponding methods to reduce the initialization sensitivity.

The remainder of this paper is organized as follows. In the section “Materials and Methods”, the proposed MSLCV model and two classical region-based active contour models are described in detail. These two classical region-based active contour models are the bases of the MSLCV model. Numerous experiments and comparisons are shown in the “Results” section. We analyze and discuss several key implementation details and improvement methods that play important roles in the accuracy and efficiency of segmentation in the “Discussion” section. Finally, in the “Conclusions” section, we summarize our work and give some directions for further research.

## Materials and Methods

### 2.1 C-V Model

Chan and Vese [16] proposed a two-phase piecewise constant model (C-V model) by simplifying Mumford and Shah’s model [36] and combining the level set method. For a given grayscale image 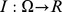, and a closed curve 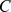, the energy function is defined as follows:
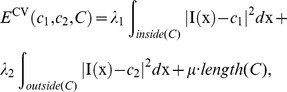
(1)where variable 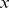 in 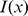 refers to a point in 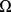, and 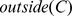 and 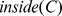 accordingly refer to regions outside and inside contour 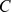, respectively. 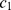 and 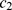 are two constants used to evaluate the image intensity of 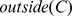 and 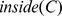. Parameters 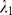, 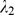 and 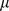 are non-negative constants in which 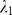 and 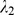 control the image data’s driving force inside and outside contour 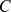, respectively, and 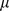 controls the smoothness of the contour. In equation (1), the first two terms use region-based global information for form fitting energy, which is called global fitting energy.

After minimizing the energy function in equation (1) and using the zero level set to express contour 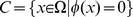 in the level set method, we can obtain 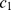 and 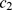 and the variational level set equation as follows:
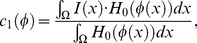
(2)

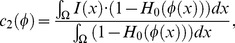
(3)

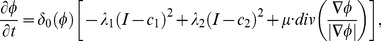
(4)where 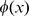 represents the level set function, 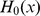 is the Heaviside function, 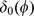 is the Dirac function, and the derivative of 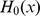 is 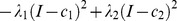, called the data fitting term, which controls the evolution of the curve. 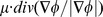, called the arc length term or regular term, determines the smoothness of the curve. The limitation of this model is that because 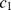 and 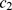 are acquired using region-based global information without considering localized image information, 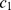 and 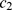 might disappear from the original data when the image intensity is inhomogeneously distributed, resulting in segmentation error.

### 2.2 Localized Region-based Active Contour Model

To overcome the difficulty that the global region-based active contour model has with processing images with inhomogeneously distributed intensity, S. Lankton *et al*. [19] proposed a localized region-based active contour model that allowed any global energy formula based on regions to be rewritten into localized form, thus segmenting these images more efficiently and precisely using localized information. The basic idea of this model is that the localized energy of every point on the curve is calculated separately. To optimize the localized energy, every point is considered separately and moves toward the minimized energy calculated for the point’s localized region. Each point’s localized neighborhood is segmented into local inside and local outside by the evolving curve. Then, energy optimization is realized by a localized region fitting model.

To define the localized region for each point on the curve, an eigenfunction is defined as [19]:
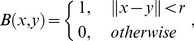
(5)where 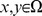 represent each point as separate space variables, and 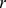 represents the radius parameter. When point 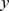 is in the circle centered at 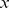 and with a radius of 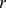, the value of the function is 1, otherwise 0.

The eigenfunction 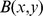 is adopted to acquire the average intensity inside and outside the localized region of point 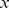 on the contour, respectively denoted by 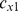 and 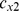:
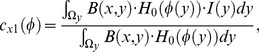
(6)

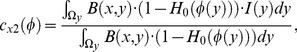
(7)where 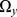 is the local region defined by 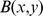. After applying the localized framework into the C-V model, the localized version of the energy function and the curvature flow for point 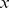 are, respectively:



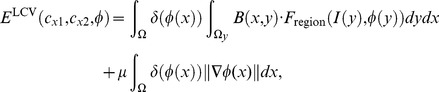
(8)

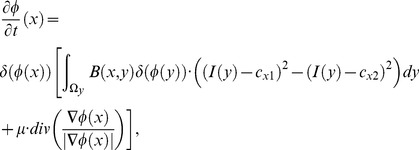
(9) where 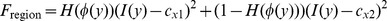 represents the region-based force, 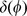 is the Dirac function, 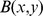 is the above-mentioned eigenfunction, 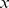 is a global point in the whole image, and 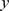 is a local point in the local region, which is the circle centered at 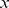 and with a radius of 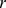. Compared with equation (4), here, 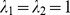. 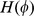, compared with 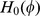 mentioned above, is only a difference of definition of inside and outside the region. In the localized version, the energy is minimized when every point on the curve has moved such that its corresponding localized inside and outside region is the best estimate of localized average 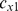 and 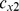.

Here, we call this localized C-V model the LCV model, which segments images with inhomogeneously distributed intensity better than does the C-V model. However, for images for HIFU therapy with extremely low SNR, low contrast and blurry boundaries, the model still easily produced boundary leakage and excessive contraction. Moreover, because the LCV model applied the C-V model on every point on the curve separately, it resulted in a large amount of calculations and became time-consuming.

### 2.3 Multi-Scale and Shape Constrained Localized Region-based Active Contour Model

To overcome the limitations of the localized region-based active contour model, we propose an accurate and efficient multi-scale and shape constrained localized region-based active contour model, called the MSLCV model, which improves segmentation accuracy and efficiency, avoids boundary leakage and excessive contraction and reduces segmentation time. By incorporating a shape constraint, we obtain more accurate segmentation for uterine fibroid HIFU ultrasound images, and by using a multi-scale segmentation method, we improve the segmentation efficiency.

#### 2.3.1 Shape Constrained Localized Region-based Active Contour Model

Traditional shape constrained models always need specified training followed by a complex matching process of shifting and stretching transformations [29], which is complicated and time-consuming. However, ultrasound imaging, due to its low SNR, low contrast and blurry boundaries, requires a good initial contour to obtain correct segmentation. Because uterine fibroids usually approximate elliptical shapes, we can set an ellipse of suitable size as an initial contour. Based on the LCV model, we utilized this initial contour as a simple and effective shape constraint to avoid boundary leakage and excessive contraction during the segmentation process. This shape constraint was incorporated into the level set framework of the LCV model, and accurate segmentation results were acquired in the experiments. Here, we call our proposed shape constrained localized region-based active contour model the SLCV model.

The basic idea is to add shape constraint energy to the process of separately calculating each point’s localized energy on the curve. The shape constraint energy is acquired by a function of the nearest distance between the point and the initial contour. We propose the following total energy function of the SLCV model by incorporating the shape constraint as:
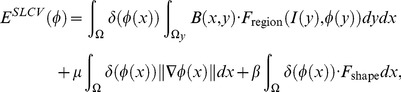
(10)where



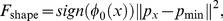
(11)

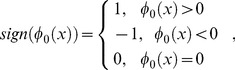
(12) where 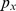 is the location of point 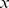 on the current contour in the image, 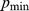 is the location of the nearest point on the initial contour 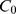 to point 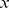, 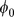 is the level set representation of the initial contour 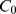, 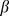 is a positive constant determining the shape constraint force, and 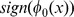 is a function determining the direction of the shape constraint force to move it towards the initial contour. Localized average values 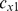 and 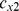 remain the same as in equations (6) and (7). In equation (10), the first term is the data fitting term, the second term is the arc length term, and the third term is the incorporated shape constraint term that forms the force toward the initial contour when the curve deforms in the evolution process.

By minimizing the energy function of the SLCV model, the corresponding level set evolution equation is:
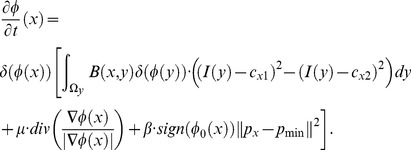
(13)



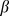
 in equation (10) and equation (13) decides the driving force of the shape constraint in the segmentation. When it approaches a maximum, the initial contour almost ceases to evolve, and the constraint degrades to non-existent when it approaches a minimum. 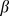 can be selected according to the quality of the images to be segmented.

In experiments, the Heaviside function 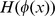 used to express the inside of contour 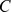 is defined using a smooth version as [19]:
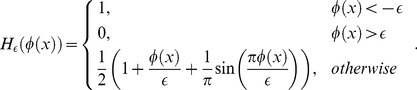
(14)


The smooth version of the Dirac function, 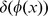, used to mark nearby regions of the curve, is obtained through derivation of 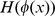:
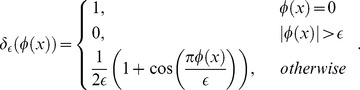
(15)


Thus, the level set evolution equation is estimated as:
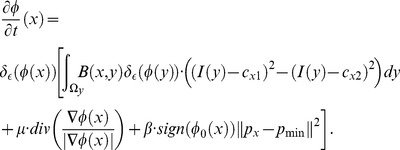
(16)


**Figure 2:**
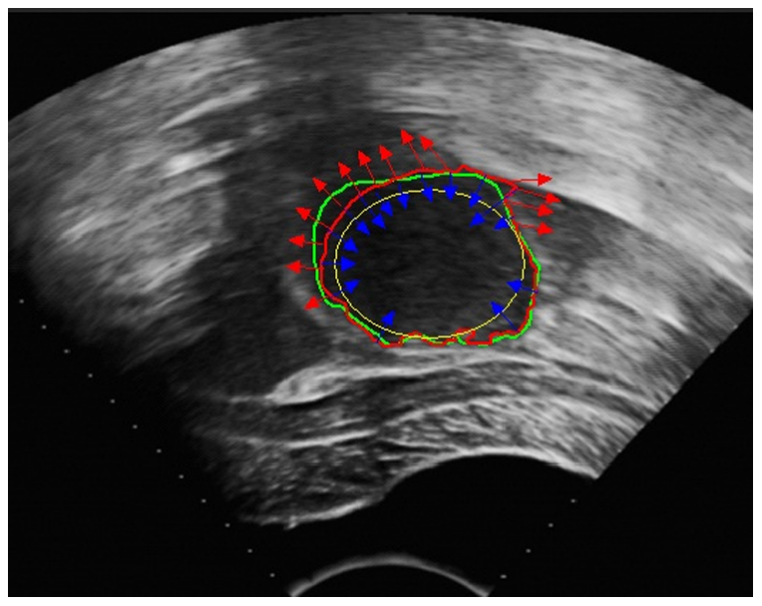
The shape constraint’s effect of avoiding boundary leakage. The yellow curve represents the initial contour, the red curve represents the contour in the process of evolution, and the green curve represents the contour drawn manually by the specialist. The red arrows display the forces of the LCV model near regions of easily occurring boundary leakage, and the blue arrows represent the shape constraint forces pointing toward the direction of the initial contour.

This SLCV model has a natural shortcoming in that it relies on the quality of the initial contour. As uterine fibroids typically approximate elliptical shapes, in the experiments, we set ellipses of different sizes as the initial contours for the segmentation of uterine fibroids. In the discussion, we have also proposed some methods to reduce the initialization sensitivity. In the experiments, we normalized the region-based energy and then used it together with the arc length term and shape constraint term to act on the evolution of the curve. Figure 2 illustrates that the shape constraint force makes a difference in the segmentation of HIFU ultrasound images of uterine fibroids, effectively avoiding boundary leakage.

#### 2.3.2 Multi-Scale Segmentation

To overcome the shortcomings of the SLCV model of large amounts of computation and a time-consuming segmentation process, we propose the MSLCV model, which combines a multi-scale segmentation algorithm with our proposed SLCV model and incorporates a multi-scale concept into the process of evolving the level set curve and effectively reduces the calculation time. The basic idea is, in the process of curve evolution, we first use a Gaussian pyramid to decompose the ultrasound image into different scale images and then perform coarse segmentation on the coarse-scale image using the SLCV model instead of directly using the original-size images. Then, we adopt the segmentation result as an initial contour for the fine-scale image, thus gradually optimizing the contour and reaching the final segmentation result. Because the amount of calculation is greatly reduced by using coarse segmentation on the coarse-scale image while obtaining an essentially correct result that is used as an initial contour for further segmentation, it takes only a few iterations to obtain satisfactory results. Thus, the MSLCV model reduces segmentation time while maintaining the accuracy of the segmentation results.

**Figure 3:**
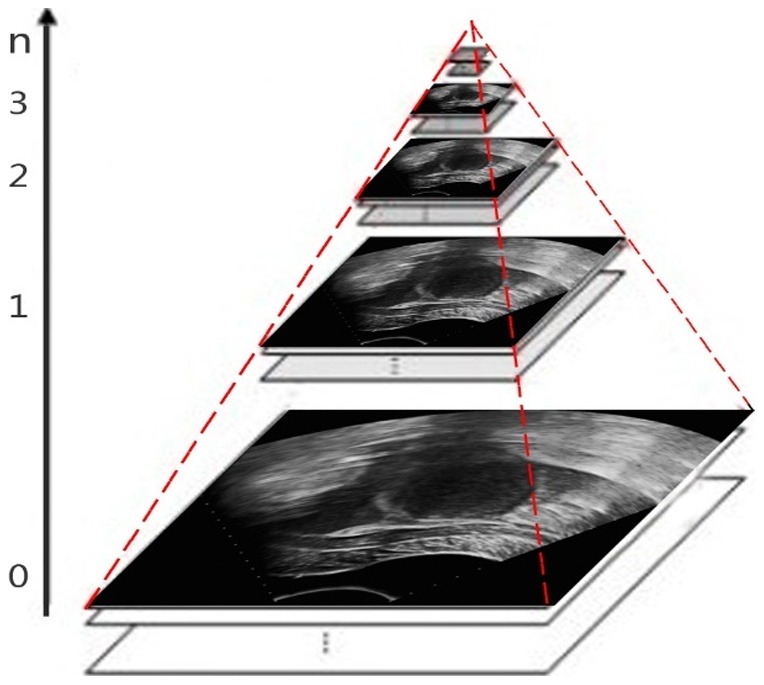
Decomposition images of the Gaussian pyramid. 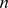
 represents for scale of Gaussian pyramid.

Let us suppose that the size of an image is 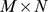, and 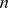 represents the scale. Now that 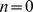 represents the original image, the image with size 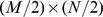 represents layer 1, …, the image with size 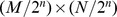 represents layer 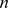, and the Gaussian pyramid formed by decomposition is as shown in Figure 3.

The original size of the ultrasound images we used in the experiments is 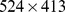. To analyze accuracy and calculation time using different scales, we segmented HIFU ultrasound images with different scales and obtained Table 1 through our experiments as follows.

**Table 1:** Accuracy and calculation time under different scales (iterations = 300).

Layer 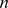	Size	Localized radius	DSC	Calculation time (s)
0	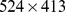	40	0.96	81.13
1	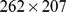	30	0.96	25.84
2	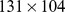	15	0.95	5.05
3	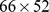	8	0.93	1.76

According to Table 1, the DSCs that represent the segmentation accuracy of layer 0, layer 1 and layer 2 are nearly the same, while for layer 3, the DSC decreases due to a large loss of information that results in the images’ becoming overly vague. In regard to the calculation time, the segmentation time drops quickly with increasing numbers of layers and decreasing image size. The segmentation time for layer 2 dropped to less than 10 seconds.

Considering accuracy and time of segmentation, we chose the image from layer 2 as the input image for coarse-scale segmentation, and the original image for fine-scale segmentation. Because of the difference in the initial contour and the different results for the two segmentations, the parameter settings are also different. In the first segmentation, the initial contour is manually initialized using an ellipse. To overcome initialization sensitivity, the weight of the shape constraint is small and the localizing radius is large, whereas in the second segmentation, the weight of the shape constraint is large and the localizing radius is small. The process for the MSLCV algorithm can be described as Table 2:

**Table 2:** The process the MSLCV algorithm.

Algorithm : MSLCV
1:** The first segmentation:**
2: Initialize contour as 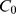 .
3: Assume the size of original image 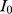 is 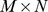 _,_ get the 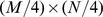 image 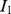 and the corresponding contour 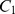 by Gaussian Pyramid, initialize level set function 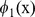 from 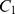 by SDF (Signed Distance Function).
4:** while** not meet the iteration stop condition **do**
5: evolve the curve 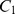 on image 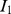 by evolving 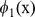 using the evolution equation (14)
6:** end while**
7: get the first segmentation result 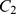 and 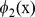 , 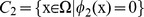 .
8:** The second segmentation:**
9: get 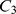 from 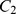 after interpolation and enlargement to the size of 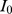 and corresponding 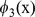 .
10:** while** not meet the iteration stop condition **do**
11: evolve the curve 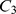 on image 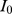 by evolving 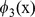 using the evolution equation (14)
12:** end while**
13: get the second segmentation result 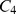 and 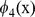 , 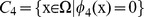 as the final result.

## Results

We conducted experiments on a desktop with an Intel CPU of Core Dual-core E7500 2.93 GHz, 2 GB RAM, Windows XP 32-bit and Matlab 2012a.

### 3.1 Segmentation of Synthetic Images

**Figure 4:**
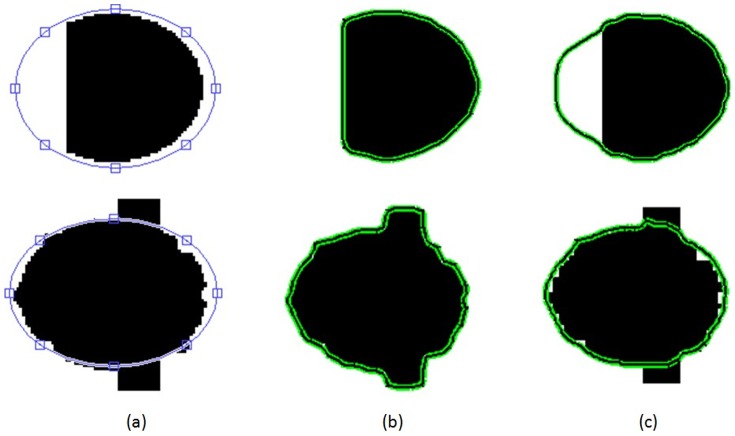
Segmentation of two synthetic images. Column (a) is initialization, column (b) is the segmentation results without shape constraint and column (c) is the segmentation results with shape constraint. Comparing column (b) with column (c), we can see that the incorporation of the shape constraint maintains an approximately elliptical contour in the final segmentation results in the segmentation of images with information loss or severe noise near the target region.

We first used synthetic images to test the effects of the shape constraint proposed in this work. When initializing contours using an ellipse on the synthesized images shown in Figure 4, we utilized a shape constraint so that the final segmentation would retain an approximately elliptical contour. However, without a shape constraint, common segmentation results will stall at clear edges and cannot maintain the true shape of the target. The experiment indicates that boundary leakage and excessive contraction will not occur in the segmentation of images with information loss or severe noise near the target region when a shape constraint is introduced to maintain the true shape of the target.

### 3.2 Segmentation of Uterine Fibroids in HIFU Ultrasound Images

In this section, we tested our proposed MSLCV model on uterine fibroids in ultrasound images for HIFU therapy and compared it with the SLCV model, which is a version of the MSLCV model without multi-scale segmentation, and other well-known methods that are suitable for ultrasound images with inhomogeneous intensity. All of the HIFU ultrasound images of uterine fibroids used here came from the HIFU center of The Second Affiliated Hospital of Chongqing Medical University.

**Figure 5:**
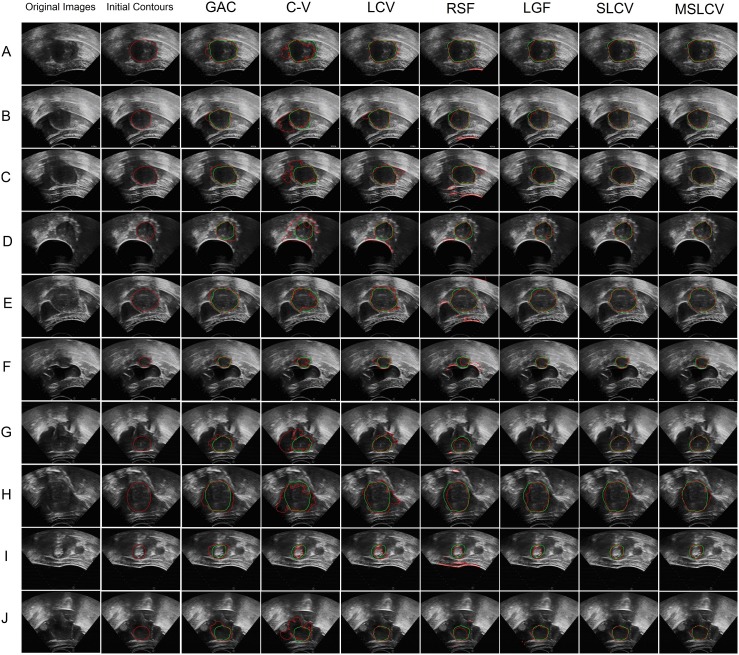
Comparison of the MSLCV and SLCV with five other well-known methods by applying them to segment 10 typical ultrasound images (A-J) of uterine fibroids for HIFU therapy. Columns 1 and 2 are the original images and the initial contours. Columns 3 to 9 respectively show the segmentation results for GAC [13], C-V [16], LCV [19], RSF [18], LGF [38], SLCV and MSLCV. The green curves are manual segmentation results by the specialist as ground truth, and the red curves are the final segmentation contours from these methods.

For ease of statistical analysis, the resolution of each image was adjusted to 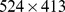. Using the software mentioned in [37], we compared the MSLCV model and the SLCV model with 5 other well-known methods on their performance in segmenting UFUS images for HIFU therapy, including an edge-based active contour model (GAC; geodesic active contours [13]) and region-based active contour models (C-V [16], LCV [19], RSF (region-scalable fitting) [18], and LGF (local Gaussian fitting) [38]). Figure 5 presents the experimental results from MSLCV, SLCV and the other five 5 methods when they were used to segment 10 typical ultrasound images of uterine fibroids for HIFU therapy. According to the images’ blurry boundaries, the HIFU ultrasound images of uterine fibroids are categorized into good, fair, and poor groups. Images A and B, with relatively clear boundaries, belong to the good group; images C, D, E and F, with blurry boundaries in some regions, belong to the fair group; and images G, H, I and J, with extremely blurry boundaries and low contrast, belong to the poor group. In the experiments, we set an ellipse of suitable size as the initial contour. The experimental results illustrate that MSLCV and SLCV can achieve more accurate segmentation results than the other 5 methods while avoiding boundary leakage and excessive contraction, even for the images in the poor group.

To more precisely estimate the quantitative comparison between the segmentation results and manual segmentation by a specialist, we adopt the Dice similarity coefficient (DSC) [39] and the mean sum of square distance (MSSD) [37] as standards. The Dice similarity coefficient is defined as:
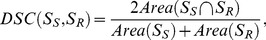
(17)where 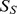 and 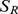 represent segmentation results and ground truth, respectively. The closer the value of the DSC is to 1, the better the segmentation result is.

The mean sum of square distance is defined as:
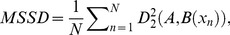
(18)

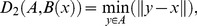
(19)where 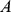 and 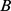 are the reference contour and the result contour of our algorithm, respectively, and 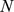 is the size of the result contour. The closer the value of the MSSD is to 0, the better the segmentation result is. We obtained table 3 via quantitative comparison of the MSLCV and the SLCV with the other five methods.

**Table 3:** Quantitative comparison of our method with the other five well-known methods using the Dice similarity coefficient (DSC) and the mean sum of square distance (MSSD) standards from the test images in Figure 5.

		GAC	C-V	LCV	RSF	LGF	SLCV	MSLCV
A	DSC	0.90	0.80	**0.95**	0.92	0.93	**0.95**	**0.95**
	MSSD	187.98	615.70	37.22	1738.96	33.02	28.89	**20.50**
B	DSC	0.91	0.70	0.92	0.86	0.93	**0.97**	0.96
	MSSD	80.98	1608.76	154.27	2165.74	44.21	**7.77**	12.38
C	DSC	0.88	0.75	0.89	0.81	0.94	**0.96**	0.95
	MSSD	84.93	1068.30	391.88	2921.86	65.3	**15.05**	16.87
D	DSC	0.88	0.50	0.83	0.88	0.94	0.94	**0.96**
	MSSD	114.23	1270.8	1231.20	1229.52	45.57	17.81	**10.38**
E	DSC	0.92	0.88	0.92	0.87	0.93	**0.95**	**0.95**
	MSSD	71.23	177.42	115.73	3052.14	52.79	**23.82**	24.45
F	DSC	0.89	0.70	0.84	0.50	0.89	**0.91**	**0.91**
	MSSD	48.91	192.44	93.16	1925.36	30.99	**16.71**	25.67
G	DSC	0.85	0.53	0.84	0.83	**0.95**	**0.95**	0.94
	MSSD	161.43	1711.53	373.05	211.47	**17.17**	17.40	19.08
H	DSC	0.89	0.73	0.85	0.89	0.90	0.92	**0.94**
	MSSD	194.69	800.51	646.14	952.10	203.53	90.37	**71.86**
I	DSC	0.78	0.63	0.55	0.50	0.69	0.85	**0.88**
	MSSD	207.86	217.14	318.28	4064.26	1832.40	49.11	**39.52**
J	DSC	0.77	0.54	0.94	0.82	0.91	0.94	**0.96**
	MSSD	322.05	1587.29	16.55	475.11	366.10	13.29	**10.87**
Averagevalue	DSC	0.87	0.67	0.85	0.79	0.90	0.93	**0.94**
	MSSD	147.43	924.99	337.75	1873.65	269.11	28.02	**25.16**

The number of iterations = 400. In the first and second segmentations of the MSLCV, the numbers of iterations were 350 and 50, respectively. Text in bold indicates the best performance for a specific image.

**Figure 6:**
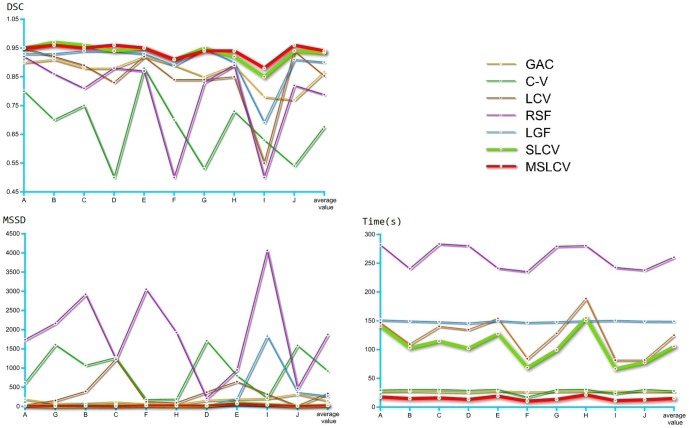
Average value Comparison comparison of DSC, MSSD and calculation time for segmentation results of GAC [13], C-V [16], LCV [19], RSF [18], LGF [38], SLCV and MSLCV by applying them on segmenting ultrasound images (A-J).

Meanwhile, in table 4, we compared the calculation times of these methods for the segmentation of UFUS images. For ease of comparison and full evolution of the curves, we set the number of iterations at 400. In Figure 5 and Table 3, we can see that the accuracy of the segmentation results for the SLCV and the MSLCV are very similar. The drop in the quality of the segmentation results when using MSLCV is almost negligible, while the segmentation efficiency is greatly improved by using the multi-scale algorithm, as shown in Table 4. This result confirms the practicability of the multi-scale segmentation algorithm. As shown in Figure 6, the MSLCV model (the red line) achieves the best performance in the segmentation of HIFU ultrasound images of uterine fibroids when considering the overall evaluation of the of the DSC, MSSD and calculation time.

**Table 4:** Calculation time (in seconds) for each method.

	GAC	C-V	LCV	RSF	LGF	SLCV	MSLCV
A	27.20	29.48	146.70	281.90	151.52	140.96	**17.69**
B	27.62	30.35	109.45	240.43	149.65	103.25	**14.97**
C	26.62	30.46	140.32	283.49	148.29	114.69	**16.03**
D	25.75	29.05	134.57	280.44	146.11	102.31	**13.46**
E	28.11	30.70	154.00	241.39	150.28	127.69	**19.17**
F	26.68	17.37	84.22	235.39	147.10	68.68	**10.51**
G	27.16	30.24	128.15	279.31	148.21	99.32	**13.63**
H	27.80	30.71	188.57	280.47	150.11	155.10	**21.41**
I	27.76	23.66	81.62	242.55	150.69	66.80	**11.30**
J	27.39	30.49	81.64	238.12	149.37	77.63	**12.81**
Average time	27.21	28.25	124.92	260.35	149.13	105.64	**15.10**

The number of iterations = 400. Text in bold indicates the best performance for a specific image.

## Discussion

### 4.1 Setting the Parameters

In equation (10), 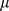 and 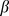 are two important parameters. 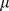 decides the smoothness of the curve, and if 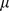 is too small, it will result in some independent points in the segmented image with substantial noise. Thus in HIFU ultrasound images with considerable noise, we usually choose a relatively large value for 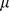 as the weight of regular term. 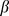 decides the value of the shape constraint forces in the segmentation. If its value is too large, the initial contour will evolve very little if at all; if it is too small, the proposed model will be degraded without shape constraint. In fact, 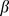 should be chosen according to the quality of the images to be segmented. It can be a relatively small value if the image has clear edges and little noise; if the opposite is true, 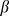 should be a relatively large value, thus enhancing the effect of the shape constraint. Meanwhile, the closer the initial contour is to the true contour of the target region, the larger 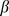 should be. In the experiments, we choose 0.2 for 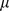 and 0.5–0.9 for 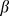 for the segmentation of HIFU ultrasound images of uterine fibroids because of the images’ quality and the uterine fibroids’ shape.

### 4.2 The Localizing Radius

**Figure 7:**
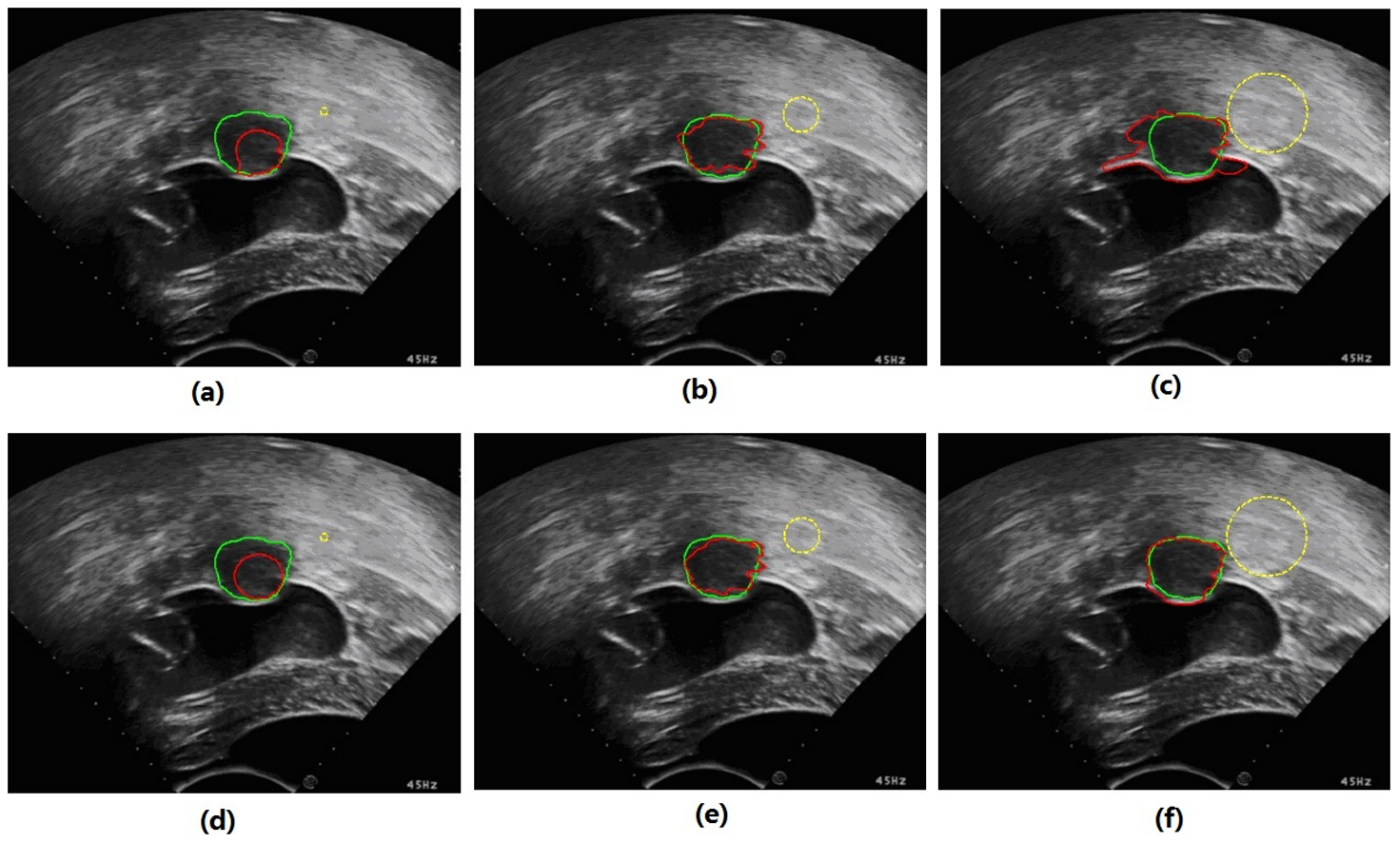
Effects of different localizing radii on the segmentation results. The first row and the second row present the segmentation results with different localizing radii using the LCV model and the MSLCV model, respectively. The green curves are manual segmentation results by the specialist, the red curves are the results from the experiments, and the yellow circles represent the size of localized regions formed by the localizing radius. The localizing radii of (a) and (d), (b) and (e), and (c) and (f) are 4, 20 and 45, respectively.

As another important parameter, the localizing radius is separately discussed here because it decides localization, thereby affecting the final segmentation results. An improper localizing radius can produce incorrect results in regions with extensive noise. Figure 7 illustrates the effects of different localizing radii on segmentation results using the LCV and MSLCV models. In Figure 7(a) and Figure 7(d), the iteration was slow under a relatively small localizing radius that led to incorrect results, while in Figure 7(c) and Figure 7(f), we observe that the LCV model easily produces boundary leakage when the localizing radius is relatively large. Because of the shape constraint and multi-scale segmentation, the MSLCV model effectively reduced the problem of boundary leakage caused by the relatively large localizing radius while worsening the difficulty in evolving the contour when the localizing radius was relatively small. Thus, it is of great importance for segmentation accuracy and efficiency to choose a suitable localizing radius.

**Figure 8:**
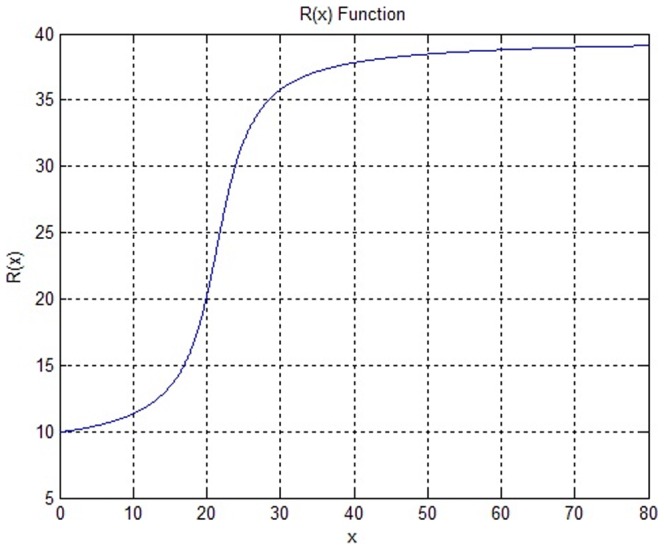
Illustration of the adaptive localizing radius function 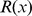 varies with 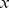,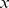 represents for scale of initial contour and is described in (21) detailedly.

S. Lankton *et al*. [19] discussed the effects of localizing radius in detail and noted that the localizing radius should be chosen according to the scale of the target region and the presence and proximity of surrounding noise, but they did not give a method for adaptive selection of the localizing radius. We do so by making use of well-initialized contours. Because difference of the size of the target region may be substantial, to automate the selection of the localizing radius, we connect the selection to the size of the well-initialized contour. We take a proportion of the sum of the difference between the maximum and minimum values on the 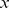 axis and 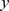 axis, respectively, of the initial contour in the image as an input parameter of the localizing radius’ adaptive selection function. For example, if we utilize an ellipse to initialize the contour, we take a proportion of the sum of the major and minor axes of the ellipse. The localizing radius’s adaptive selection function 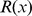 is defined as:
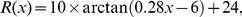
(20)where
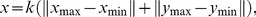
(21)where 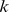 is a coefficient that controls the proportion, which is usually set as 0.25. 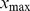 and 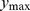 respectively, are the maximum values of the initial contour on the 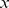 axis and 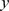 axis, while 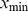 and 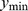 are the minimum values. Figure 8 demonstrates that the function 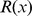 effectively avoids the problem of the localizing radius being too large or too small when the segmentation target is too large or too small. We set a range of 10 to 40 for the localizing radius according to the size of the uterine fibroids in the HIFU ultrasound images and experimental results to avoid the lack of evolution of the contour that occurs when the localizing radius is too small and the boundary leakage and greatly increased calculation time that occur when the localizing radius results is too large. In the multi-scale segmentation, we set a larger 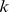 in the first segmentation to obtain a larger localized radius, faster convergence and weaker initialization sensitivity, and set a smaller 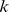 on the second segmentation to reduce the calculation time.

### 4.3 Reducing initialization sensitivity

**Figure 9:**
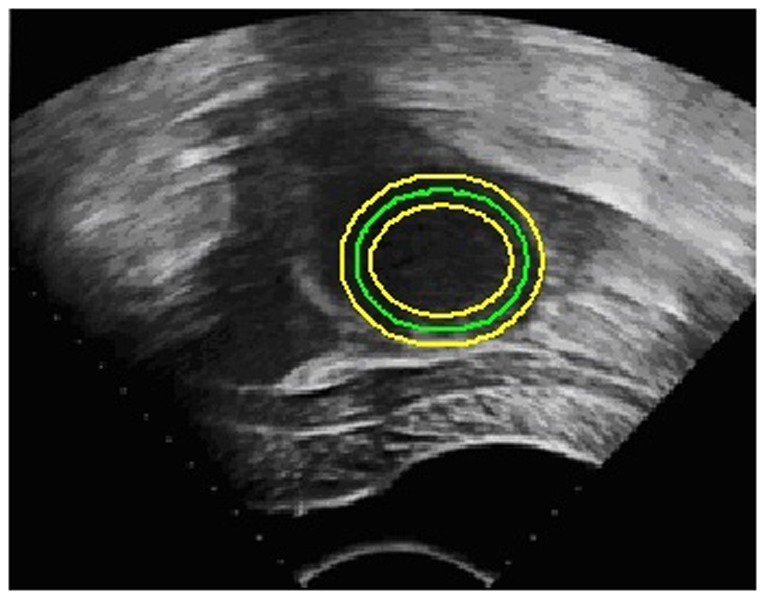
Zero narrow band. The green curve represents the initial contour 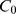, and the two yellow curves 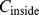 and 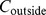 are the edges of the narrow band.

The effect of the incorporated shape constraint relies on the initial contour. To reduce initialization sensitivity, we consider using a zero narrow band that is generated around the zero level set created by the initial contour. The shape constraint is ignored within the zero narrow band. As shown in Figure 9, the green curve represents the initial contour, 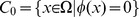, the yellow curve inside the green curve is 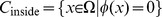, and the yellow curve outside the green curve is 
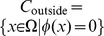
. The width of the zero narrow band is represented by 2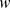. Ignoring the constraint within the zero narrow band reduces the effect of the shape constraint around the initial contour and thus reduces the initialization sensitivity. In the multi-scale segmentation, for the first segmentation, we use the zero narrow band to reduce the initialization sensitivity due to the manually initialized contour, while on the second segmentation, we do not use the zero narrow band because the coarse contour has already been confirmed.

## Conclusions

In this paper, an accurate and efficient multi-scale and shape constrained localized region-based active contour model, called the MSLCV model, has been proposed to perform semi-automatic segmentation of uterine fibroid in ultrasound images for HIFU therapy. By incorporating a new shape constraint into the localized region-based active contour, we have obtained a more precise segmentation result, avoiding the problems of boundary leakage and excessive contraction due to the low SNR, weak boundaries and intensity inhomogeneity of HIFU ultrasound images. Further, to overcome the shortcomings of the large computation time and the time-consuming nature of the segmentation process in the localized region-based active contour model, we have proposed a multi-scale algorithm that greatly improves the segmentation efficiency. Meanwhile, to solve the problem of the selection of localizing radius and initialization sensitivity, we have discussed and analyzed the adaptive selection of the localizing radius and the formation of a zero narrow band. Compared with other well-known methods, the MSLCV model provides more accurate and efficient segmentation results that are closer to the manual segmentation results obtained by a specialist. In future work, we will further improve the segmentation efficiency by GPU acceleration and study the adaptive change of the shape constraint’s effect according to the quality of the HIFU ultrasound images of uterine fibroids to acquire better segmentation results.
